# Supplementary figures and images for: In Vitro Antibiofilm Activity of Resveratrol against Aeromonas hydrophila
Source: Antibiotics (Basel). 2023 Mar 31;12(4):686. doi: 10.3390/antibiotics12040686 (PMC10135085; doi:10.3390/antibiotics12040686)

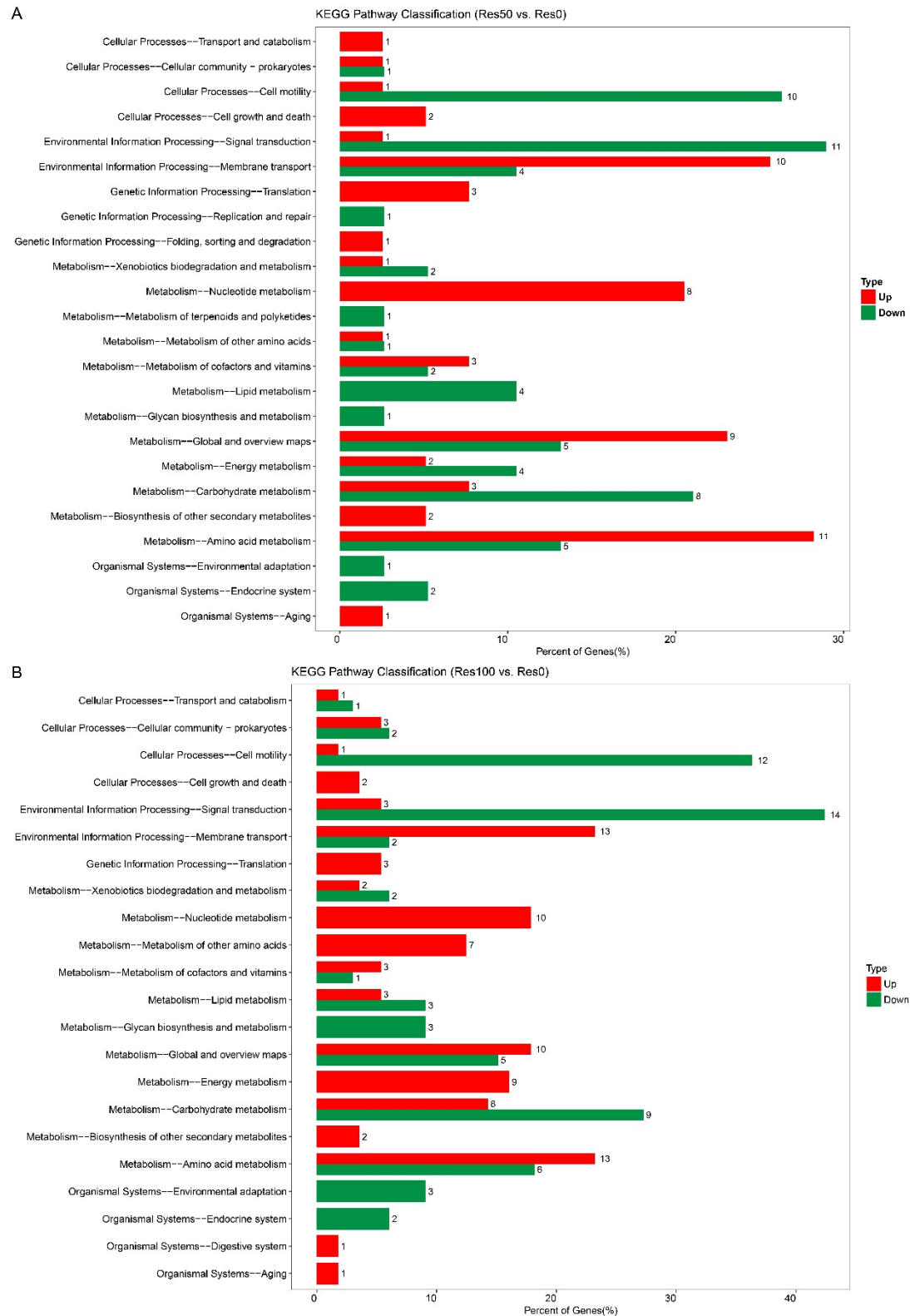

Figure S1. KEGG pathway classification. (A) Res 50 vs. Res 0 groups. (B) Res 100 vs. Res 0 groups.

Supplement: Supplementary file 1 [file antibiotics-12-00686-s001.zip › antibiotics-2263886-supplementary.pdf]
